# Medical student and medical school teaching faculty perceptions of conflict of interest

**DOI:** 10.1186/s13104-017-2596-7

**Published:** 2017-07-11

**Authors:** Nicholas S. Andresen, Tyler S. Olson, Matthew D. Krasowski

**Affiliations:** 1 University of Iowa, Carver College of Medicine, 200 Hawkins Drive, Iowa City, IA 52242 USA; 2 Department of Pathology, University of Iowa, Carver College of Medicine, 200 Hawkins Drive, Iowa City, IA 52242 USA

**Keywords:** Conflict of interest, Education and training, Continuing medical education, Medical education, Disclosure

## Abstract

**Background:**

Attitudes towards conflict of interest (COI) and COI policy are shaped during medical school and influence both the education of medical students and their future medical practice. Understanding the current attitudes of medical students and medical school teaching faculty may provide insight into what is taught about COI and COI policy within the ‘hidden’ medical curriculum. Differences between medical student and medical school teaching faculty perceptions of COI and COI policy have not been compared in detail. The authors surveyed first year medical students and medical school teaching faculty at one academic medical center.

**Results:**

The response rate was 98.7% (150/152) for students and 34.2% (69/202) for faculty. Students were less likely than faculty to agree that lecturers should disclose COI to any learners (4.06 vs. 4.31, p = 0.01), but more likely to agree that COI disclosure decreases the presentation of biased material (3.80 vs. 3.21, p < 0.001). Student and faculty responses for all other questions were not different. Many of these responses suggest student and faculty support for stronger COI policy at academic medical centers.

**Conclusions:**

Students and faculty perceptions regarding COI and COI policy are largely similar, but differ in terms of the perceived effectiveness of COI disclosure. This study also suggests that medical students and medical school teaching faculty support for stronger COI policy at academic medical centers.

## Background

Managing conflicts of interest (COI) and creating effective COI policy is a difficult, yet important, task for academic medical centers as they perform the missions of patient care, biomedical research, and medical education. Weak COI policies at academic medical centers can threaten the integrity of these missions, and may do so with lasting effects. For example, medical school graduates from institutions with stronger COI policies demonstrate more evidence-based and cost-effective prescribing habits [1].

As a result of these concerns, there has been a recent, international scrutiny of COI policies at medical schools and academic medical centers. Authors from Canada and several European countries have published studies highlighting the generally weak COI policies seen at medical schools within their countries, calling for change [2–4] This focus on COI policy has been even more poignant within the United States where national organizations such as The American Medical Student Association (AMSA) and Pew Charitable Trust have recently called for and taken steps towards encouraging more restrictive COI policies [5, 6]. For example, the Pew Charitable Trust has published comprehensive recommendations on COI policy, including mandatory disclosure of COI during all lectures, prohibition of any gifts from industry representatives, and limiting the relationships that employees or students may have with industry [6]. Based upon these criteria, AMSA has created a national scorecard to assess COI policies at all US medical schools [5]. Coinciding with this increased focus on COI, many medical schools and academic medical centers have strengthened their COI policies over the past 10 years [5]. However, these efforts have largely been top-down, coming from regulatory bodies, national organizations, and administrators. Some have questioned the need for strengthening these policies, citing a lack of evidence supporting the need for stronger COI policy and the potential harms of restricting relationships with industry [7].

There has been little investigation into the perception that medical students and medical school teaching faculty have regarding COI. Several studies have assessed student opinions of COI [8–10] but no study has investigated both faculty and student perceptions of COI policy simultaneously.

These perceptions are important for two reasons. First, the differences between student and faculty training may provide insight into how the process of medical training alters attitudes towards COI and COI policy. Specifically, first year medical students may be largely unexposed to the potential COI that exist within healthcare, while faculty may have had experience with COI that has shaped their perceptions. Second, student and faculty opinions are important because they reflect what may be taught in the ‘hidden curriculum’ of medical schools. The perceptions of COI held by faculty may be reflected in statements and actions that are not always congruent with institutional COI policy.

To develop a better understanding of student and faculty perceptions of COI and COI policy at one academic medical center we gave the same 10-question survey to both students and faculty.

## Methods

This study occurred at the University of Iowa Carver College of Medicine (CCOM). CCOM requires COI disclosure slides (‘second slides’) in all preclinical lectures and has a COI policy that received a ‘B’ on AMSA’s 2014 Just Medicine Scorecard [11]. All faculty at CCOM, as part of employment requirements, have completed COI online training and additionally provide annual disclosures of COI.

We developed a ten-question survey to assess medical student and medical school teaching faculty attitudes towards COI and COI policy (Table 1). Survey questions were adapted from three prior studies that have yielded important findings related to conflict of interest. [8, 9, 12] We selected questions that we believed would provide the most comprehensive information possible within a ten-question survey. All questions were on a five point Likert scale (1 = strongly disagree, 2 = disagree, 3 = neutral, 4 = agree, 5 = strongly agree). The same version of this survey was given to all first year medical students and teaching faculty who lecture to first or second year medical students. The survey was not pilot-tested at our institution before being distributed.

**Table 1:** Survey given to both medical students and medical school teaching faculty

*Question 1* It is acceptable for healthcare professionals to receive gifts or food from pharmaceutical or medical device companies
*Question 2* It is acceptable for medical students to receive gifts or food from pharmaceutical or medical device companies
*Question 3* If I accept gift or food from pharmaceutical or medical device company representatives, I will be more likely to prescribe, use, or recommend those company’s products now or in the future
*Question 4* Healthcare professionals who accept gifts or food from pharmaceutical or medical device company representatives will be more likely to prescribe, use, or recommend those company’s products
*Question 5* Pharmaceutical and medical device company representatives should be prohibited from meeting with healthcare professionals
*Question 6* Pharmaceutical and medical device company representatives should be prohibited from meeting with medical students
*Question 7* Medical schools should require educators to disclose to learners any monies received from pharmaceutical or medical device companies for speaking, consulting, travel, or research
*Question 8* Medical educators who disclose their potential conflicts of interest are less likely to present biased material than those who do not disclose
*Question 9* Healthcare providers should disclose to patients any monies received from pharmaceutical or medical device companies for speaking, consulting, travel, or research, if it is related to a product or device that will be used in that patient’s care
*Question 10* Healthcare providers should disclose to patients any monies received from pharmaceutical or medical device companies for speaking, consulting, travel, or research, regardless of the reason for that patient’s visit

First year medical students (n = 152) were given an electronic version of the survey during their orientation to medical school. Students were instructed that the survey was available on a digital tablet outside of their lecture hall during an afternoon of orientation. Teaching faculty were identified from a preclinical ‘lecturer roster’ provided by CCOM administrative staff. Faculty were emailed an electronic version of the survey (n = 202). Student and faculty responses were collected via secure Qualtrics Survey Software [13]. All participation was voluntary and anonymous.

Differences between student and faculty responses were analyzed by a Mann–Whitney U test, with significance defined as a P value of less than 0.05. Cross-referencing of student and faculty responses was performed based upon their response to question 1 (Tables 3, 4). Individuals who responded to question one with a Likert response of 1 or 2 were sorted into one group, while individuals who responded with a 4 or 5 were placed in a second group. Differences between grouped responses to questions three through ten were then tested with a Mann–Whitney U test, with significance defined as a P value of less than 0.05.

Mann–Whitney U tests were used instead of parametric tests because the distribution of data was non-normal. IBM SPSS Statistics 23 was used for statistical analysis [14]. The University of Iowa Carver College of Medicine Institutional Review Board (IRB) approved the study under the umbrella IRB for medical education.

## Results

A total of 150 of the 152 (98.7%) first-year medical students completed the survey. The faculty response rate was lower as 69 of the 202 (34.2%) faculty completed the survey. The mean age for students of 23.8 (SD = 2.53) years was lower than the mean age for faculty of 49.5 (SD = 10.8) years. Student and faculty did not differ by gender with 49.3% of students and 56.5% of faculty being male.

Faculty were more likely than students to agree that that medical school lecturers should be required to disclose COI to learners (student = 4.06, faculty = 4.31, p < 0.001). However, faculty were less likely to believe that disclosing potential COI would result in the presentation of less biased material (student = 3.80, faculty = 3.21, p < 0.001). Student and faculty responses were not different for any other questions.

Both medical students and faculty indicated that is unacceptable for medical students or faculty to accept gifts from pharmaceutical or medical device representatives (Table 2). Similarly, both students and faculty considered their peers to be more susceptible to influence from gifts or food then they were themselves (Table 2). Students and faculty also indicated that physicians should be required to disclose COI to patients if it is relevant to their care, but not necessarily if the conflict was not relevant to that patient’s care (Table 2). All results are summarized in Table 2.

**Table 2:** Student and faculty perceptions of conflict of interest

Statement	Students		Faculty		*p* value
	Mean	SD	Mean	SD	
*Question 1* It is acceptable for healthcare professionals to receive gifts or food from pharmaceutical or medical device companies^a^	2.03	1.05	2.20	1.09	0.25
*Question 2* It is acceptable for medical students to receive gifts or food from pharmaceutical or medical device companies^a^	2.00	1.09	2.20	1.11	0.16
*Question 3* If I accept gift or food from pharmaceutical or medical device company representatives, I will be more likely to prescribe, use, or recommend those company’s products now or in the future^a^	2.13	1.21	2.44	1.32	0.102
*Question 4* Healthcare professionals who accept gifts or food from pharmaceutical or medical device company representatives will be more likely to prescribe, use, or recommend those company’s products^a^	3.23	1.10	3.17	1.28	0.791
*Question 5* Pharmaceutical and medical device company representatives should be prohibited from meeting with healthcare professionals^a^	2.27	1.00	2.07	1.12	0.10
*Question 6* Pharmaceutical and medical device company representatives should be prohibited from meeting with medical students^a^	2.83	1.16	2.96	1.39	0.52
*Question 7* Medical schools should require educators to disclose to learners any monies received from pharmaceutical or medical device companies for speaking, consulting, travel, or research^a^	4.06	0.96	4.30	1.05	0.01
*Question 8* Medical educators who disclose their potential conflicts of interest are less likely to present biased material than those who do not disclose^a^	3.79	0.88	3.22	1.04	<0.001
*Question 9* Healthcare providers should disclose to patients any monies received from pharmaceutical or medical device companies for speaking, consulting, travel, or research, if it is related to a product or device that will be used in that patient’s care^a^	3.98	0.98	4.09	0.90	0.55
*Question 10* Healthcare providers should disclose to patients any monies received from pharmaceutical or medical device companies for speaking, consulting, travel, or research, regardless of the reason for that patient’s visit^a^	3.08	1.20	2.74	1.22	0.54

Responses were collected from 149 first-year medical students and 68 medical school teaching faculty in August 2015 at the University of Iowa Carver College of Medicine

*SD* standard deviation

^a^Responses are based upon a five point Likert scale. Response scale: 1 = strongly disagree, 2 = disagree, 3 = neutral, 4 = agree, 5 = strongly agree

Cross-referencing analysis revealed that 103 (68%) students responded to question one regarding the acceptability of receiving gifts from pharmaceutical or medical device companies with a Likert response of 1 or 2, while 17 (11%) responded with a 4 or 5 (Table 3). Cross-references of faculty responses revealed that 44 (63%) of faculty responded to question one with a Likert response of 1 or 2, while 9 (13%) responded with a 4 or 5 (Table 4). For both students and faculty, the perceived acceptability of receiving gifts from pharmaceutical or medical device representatives was linked to opinions regarding whether industry representative should have contact with healthcare professional or medical students (Tables 3, 4; Q5 and Q6). For faculty, but not students, the perceived acceptability of receiving gifts was linked to the perceived ability of these gifts to alter healthcare practice (Tables 3 and 4; Q3 and Q4). For students, but not faculty, the perceived acceptability of receiving gifts was tied to beliefs regarding situations in which COI should be disclosed (Tables 3, 4; Q7, Q9, and Q10).

**Table 3:** Cross-referencing of students perceptions of conflict of interest based upon their response to question 1

Statement	Number of students answering Q1 with a 1 or 2	Number of students answering Q1 with a 4 or 5
*Question 1* It is acceptable for healthcare professionals to receive gifts or food from pharmaceutical or medical device companies^b^	103 (68%)	17 (11%)

	Mean	SD	Mean	SD	p-value
*Question 3* If I accept gift or food from pharmaceutical or medical device company representatives, I will be more likely to prescribe, use, or recommend those company’s products now or in the future^a^	2.20	1.30	2.29	1.04	0.455
*Question 4* Healthcare professionals who accept gifts or food from pharmaceutical or medical device company representatives will be more likely to prescribe, use, or recommend those company’s products^a^	3.37	1.07	3.00	1.06	0.129
*Question 5* Pharmaceutical and medical device company representatives should be prohibited from meeting with healthcare professionals^a^	2.36	1.02	1.64	0.78	0.003
*Question 6* Pharmaceutical and medical device company representatives should be prohibited from meeting with medical students^a^	2.95	1.19	2.29	1.04	0.027
*Question 7* Medical schools should require educators to disclose to learners any monies received from pharmaceutical or medical device companies for speaking, consulting, travel, or research^a^	4.21	0.92	3.70	1.04	0.036
*Question 8* Medical educators who disclose their potential conflicts of interest are less likely to present biased material than those who do not disclose^a^	3.81	0.96	3.82	0.72	0.764
*Question 9* Healthcare providers should disclose to patients any monies received from pharmaceutical or medical device companies for speaking, consulting, travel, or research, if it is related to a product or device that will be used in that patient’s care^a^	4.12	1.00	3.52	0.79	0.004
*Question 10* Healthcare providers should disclose to patients any monies received from pharmaceutical or medical device companies for speaking, consulting, travel, or research, regardless of the reason for that patient’s visit^a^	3.26	1.22	2.52	0.94	0.022

Responses were collected from 149 first-year medical students and 68 medical school teaching faculty in August 2015 at the University of Iowa Carver College of Medicine

*SD* standard deviation

^a^Responses are based upon a five point Likert scale. Response scale: 1 = strongly disagree, 2 = disagree, 3 = neutral, 4 = agree, 5 = strongly agree

^b^Counts represent the number of students who responded to question 1 with either a 1 or 2, or with a 4 or 5 respectively. The means reported for questions 3–10 are based upon the responses from individuals within these categories

**Table 4:** Cross-referencing of faculty perceptions of conflict of interest based upon their response to question 1

Statement	Number of faculty answering Q1 with a 1 or 2	Number of faculty answering Q1 with a 4 or 5
*Question 1* It is acceptable for healthcare professionals to receive gifts or food from pharmaceutical or medical device companies^b^	44 (63%)	9 (13%)

	Mean	SD	Mean	SD	p-value
*Question 3* If I accept gift or food from pharmaceutical or medical device company representatives, I will be more likely to prescribe, use, or recommend those company’s products now or in the future^a^	2.86	1.39	1.66	0.70	0.023
*Question 4*: Healthcare professionals who accept gifts or food from pharmaceutical or medical device company representatives will be more likely to prescribe, use, or recommend those company’s products^a^	3.68	1.13	2.11	0.92	0.001
*Question 5* Pharmaceutical and medical device company representatives should be prohibited from meeting with healthcare professionals^a^	2.36	1.16	1.55	1.01	0.034
*Question 6* Pharmaceutical and medical device company representatives should be prohibited from meeting with medical students^a^	3.34	1.34	1.55	1.01	0.001
*Question 7* Medical schools should require educators to disclose to learners any monies received from pharmaceutical or medical device companies for speaking, consulting, travel, or research^a^	4.45	0.84	4.11	1.36	0.666
*Question 8* Medical educators who disclose their potential conflicts of interest are less likely to present biased material than those who do not disclose^a^	3.40	0.92	3.33	1.00	0.843
*Question 9* Healthcare providers should disclose to patients any monies received from pharmaceutical or medical device companies for speaking, consulting, travel, or research, if it is related to a product or device that will be used in that patient’s care^a^	4.11	0.86	4.00	1.11	0.898
*Question 10* Healthcare providers should disclose to patients any monies received from pharmaceutical or medical device companies for speaking, consulting, travel, or research, regardless of the reason for that patient’s visit^a^	2.61	1.12	3.33	1.22	0.134

Responses were collected from 149 first-year medical students and 68 medical school teaching faculty in August 2015 at the University of Iowa Carver College of Medicine

*SD* standard deviation

^a^Responses are based upon a five point Likert scale. Response scale: 1 = strongly disagree, 2 = disagree, 3 = neutral, 4 = agree, 5 = strongly agree

^b^Counts represent the number of faculty who responded to question 1 with either a 1 or 2, or with a 4 or 5 respectively. The means reported for questions 3–10 are based upon the responses from individuals within these categories

## Discussion

This study represents the first time that perceptions of COI have been assessed simultaneously for both students and preclinical lecturing faculty. These results are limited by the fact that they come from one institution and there was a much lower survey response rate for faculty. However, these results still provide new insight into perceptions of COI and hold some implications for COI policy at academic medical centers.

Student and faculty perceptions of COI are largely similar. This suggests that the process of medical training does not dramatically change perceptions of COI. Specifically, the first year medical students surveyed in this study may not have yet been exposed to COI within medical training and healthcare, while all faculty surveyed have likely witnessed COI during their medical training, healthcare experience, or continuing medical education courses.

The differences that did arise in student and faculty and student opinions provide further insight into how medical training may change perceptions. Namely, faculty were more likely than students to believe that disclosure should be required, but less likely to believe that disclosure would ensure the presentation of less biased material. There are several possible explanations for this finding. First, it may be that faculty have had experiences (e.g. witnessing COI influence patient care) during their medical training that has reinforced the importance of disclosing COI. Further, these experiences may have also led faculty to recognize that disclosure alone is not enough to limit the influences of COI. Second, it is possible that since we had a lower survey response rate from faculty than from students, we may have selected for faculty that recognize the importance of COI disclosure, but also its limitations. Third, it could be that faculty perceive themselves and other faculty to be more objective than they are perceived to be by their students, thus making faculty less likely to believe that disclosure will lead to the presentation of more objective information. While we cannot state with certainty the reason for these differences, these results at least suggest that faculty may have had experiences during medical training which lead them to believe that disclosure alone is not enough to temper the influences of COI.

These results also provide support for the stronger COI policies being advocated for by several authors internationally and by national organizations within the United States. Specifically, these results suggest that students and faculty are largely in favor of these policies. Both students and faculty indicated that their peers are more susceptible to the influence of COI than they were themselves. This trend has been found in the past for physicians [15] and indicates an increased need for COI education amongst students and faculty. Additionally, there was general agreement that it was unacceptable to receive gifts from industry, disclosure should be required to learners, and that disclosure to patients should even be required in certain situations.

Last, these results shed some light on the motivations for student and faculty opinions regarding COI. Unsurprisingly, both students and faculty who believe that receiving gifts in unacceptable also believe that contact with industry representatives should be limited. For faculty, the perceived acceptability of accepting gifts from pharmaceutical or medical device representatives was linked to the belief that accepting gifts has the power to alter behaviors. However, this relationship did not hold true for students, suggesting that faculty are more likely than students to consider forces of COI to alter practice patterns when forming opinions related to COI. Further, for students the perceived acceptability of receiving gifts was linked to opinions about when COI should be disclosed. This relationship did not hold true for faculty, suggesting that other factors, such as the perceived effectiveness or feasibility of such a disclosure, may have a stronger influence on beliefs regarding appropriate COI disclosure.

## Conclusions

Medical students and medical school teaching faculty have similar perceptions of COI and COI policy. However, faculty are less likely to believe that COI disclosure to learners results in the presentation of less biased material. This suggests that the experiences of faculty within medical education and medical practice may give them reason to doubt the effectiveness of COI disclosure. The attitudes of students and faculty suggest support for the stronger COI policies. Lastly, these results shed light on the different motivations that may lay behind student and faculty perceptions of COI.
